# The Influence of Individual Characteristics, Personality Traits, and Life Events on Academic Stress: A Survey among Third-Year Medical Students at Humanitas University

**DOI:** 10.1192/j.eurpsy.2025.2000

**Published:** 2025-08-26

**Authors:** D. Caldirola, S. Daccò, F. Cuniberti, V. Butti, M. Grassi, A. Spiti, G. Perna

**Affiliations:** 1 Department of Biomedical Sciences, Humanitas University, Pieve Emanuele, 20090 Milan; 2 Humanitas San Pio X, 20159 Milan; 3 IRCCS Humanitas Research Hospital, Rozzano, 20089 Milan, Italy

## Abstract

**Introduction:**

Medical students face a demanding academic journey, where stress can significantly impact their well-being and performance. Both personal and environmental factors contribute to this burden, yet research on academic stress in Italian medical schools remains limited.

**Objectives:**

We investigated the impact of various factors on perceived academic stress among third-year medical students during the pre-clinical phase of their studies.

**Methods:**

A cross-sectional, anonymous, self-reported online survey was distributed to third-year medical students at Humanitas University (Milan, Italy) over a 10-day period in April 2024. The survey included various questions on sociodemographic factors, lifestyle behaviors (e.g., sports activity, diet, smoking, alcohol binge drinking), satisfaction with sleep and sexual activities, smartphone and video game use, significant life events, and objective academic performance. Additionally, several validated questionnaires were included to assess academic stress, personality traits, psychological dispositions (e.g., proneness to boredom, procrastination, impulsivity, perfectionism, intolerance of uncertainty, worry), coping strategies, resilience, attentional control, feelings of loneliness, and perceived social support. Data were analyzed using hierarchical multiple linear regression, with a significance level at 0.05.

**Results:**

Seventy-six students (70.4%) out of 108 who provided their informed consent completed the survey; 58 (76.3%) were female and 18 (23.7%) were male, with 53 (69.7%) being Italian and 23 (30.3%) from other countries. Higher academic stress was significantly associated with the female gender, a higher burden of negative life events, and greater propensity for impulsivity and worry. In contrast, passing more exams and experiencing positive life events were associated with lower academic stress. No other statistically significant associations were found. The independent variables collectively explained about 60% of the variation in academic stress (adjusted R², p<0.001).

**Conclusions:**

Our preliminary findings provide insights into the factors influencing the multifaceted nature of academic stress. These results may help educators, administrators, and policymakers create a more supportive learning environment and offer psychological support to reduce academic stress in vulnerable students. Enhancing the psychological well-being of medical students could help prevent potential mental health conditions in this population. Further studies with larger samples and a prospective design are warranted.

**Disclosure of Interest:**

None Declared

